# Concomitant intake of high levels of monosodium glutamate and taurine induces adverse effects in the rat brain

**DOI:** 10.1016/j.toxrep.2026.102276

**Published:** 2026-05-22

**Authors:** Yasemin Atici, Makbule Beyza Sen, Atakan Saglam, Gamze Dogan, Dogan Yucel, Belma Turan

**Affiliations:** a Department of Medical Biochemistry, Lokman Hekim University, Faculty of Medicine, Ankara, Türkiye; b Department of Biochemistry, Lokman Hekim University, Faculty of Pharmacy, Ankara, Türkiye; c Department of Histology and Embryology, Ankara University, Faculty of Medicine, Ankara, Türkiye; d Department of Biophysics, Lokman Hekim University, Faculty of Medicine, Ankara, Türkiye

**Keywords:** Brain, Cortex, Energy drinks, Fast-foods, Monosodium glutamate, Oxidative stress, Taurine

## Abstract

A common food additive, monosodium glutamate (MSG), is a source of glutamate, the primary excitatory neurotransmitter in the central nervous system, and has been associated with neuronal damage at high levels. Taurine (TAU), a naturally occurring amino acid, is widely used in energy drinks alongside caffeine and sugar. Given the widespread consumption of these products, this study investigated the potential adverse effects of high levels of MSG, TAU, and their combination on systemic and brain oxidative stress/antioxidant balance, as well as cortical histology. Young male Wistar rats (10 weeks old) were randomly assigned to four groups: MSG (1 g/kg/day), TAU (300 mg/kg/day), MSG+TAU (1 g/kg/day + 300 mg/kg/day), and control (CON) group (saline), all administered via oral gavage for 21 days. While serum trace element levels (Cu, Zn, Se) remained unaffected, total oxidant status was significantly higher in the MSG and MSG+TAU groups compared to the TAU and control groups. Oxidative stress markers (8-OHdG and 4-HNE) were elevated in the serum, cortex, and hippocampus of all experimental groups, accompanied by increased total oxidant status and decreased total antioxidant status in these samples. Histological analysis revealed neuronal disorganization and pericapillary edema in the cerebral cortex. These findings indicate that excessive exposure to these compounds may disrupt redox balance and brain tissue integrity to varying degrees, with combined exposure producing more pronounced deleterious effects. Overall, these results suggest that frequent consumption of foods and beverages containing MSG and taurine, particularly at high levels, may pose significant health risks to young individuals.

## Highlights

• High-level administration of monosodium glutamate (MSG) and taurine (TAU) in rats increased oxidative stress markers in serum and the hippocampus.
• These effects were associated with cortical histopathological alterations, including neuronal disorganization and pericapillary edema.
• These findings suggest an association between increased oxidative stress and structural alterations in brain tissue under conditions of excessive MSG and TAU exposure.

## Graphical Abstract

**Figure:**
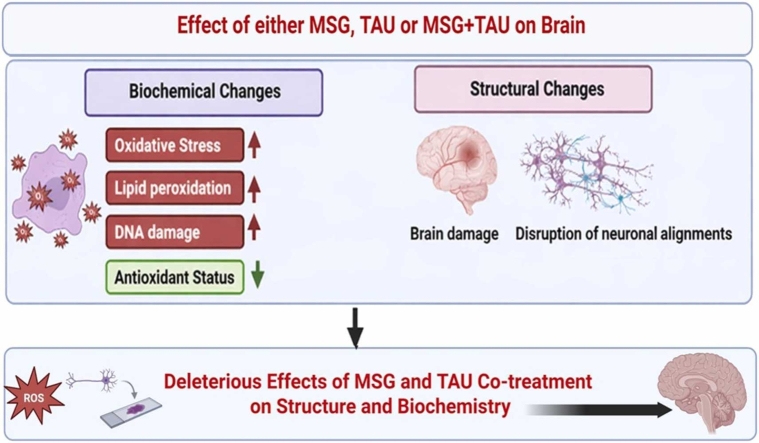

## 1 Introduction

The global consumption of fast-foods and energy drinks has risen substantially in modern societies. So, it is raising critical concerns regarding food safety and processing, particularly due to the widespread use of added ingredients and chemical additives. Consequently, ready-made foods and beverages, especially energy drinks, have become integral not only to contemporary nutrition but also to daily lifestyle patterns [1], [2], [3]. Despite their popularity, significant debate persists concerning the chemical constituents used in their formulation and preservation. Of particular concern are the potential acute and chronic adverse effects of these compounds on human health, which remain insufficiently characterized. Given the accumulating evidence that consumption of such products is disproportionately high among adolescents and young adults, these concerns underscore the potential for substantial long-term public health implications [4], [5], [6].

Taurine (TAU) is a multifunctional amino acid involved in a wide range of physiological processes. One important example of TAU action in cells is used for energy production [6], [7], [8]. The main dietary sources of TAU are protein-rich animal foods such as meat, shellfish, and dairy, and also available as a supplement, particularly to help people manage certain conditions or diseases [1], [3], [9], [10]. Given the beneficial effects of TAU in promoting many physiological functions and performance benefits in the human body, it has become increasingly popular in a variety of ready-to-eat food**s**, dietary supplements, and energy drinks. Nutritional research in the area supports the beneficial effects of TAU in promoting many physiological functions and performance benefits. Also, energy drinks often include not only TAU but also high amounts of sugar and caffeine, while they’re not only high amounts, but also frequent consumption can be harmful for our health [10], [11], [12]. In addition, as summarized in a comprehensive review, the adverse effects of energy drinks on the human body have been well documented [13]. These beverages have been associated with dehydration, sleep disturbances, and increased feelings of nervousness and tension in humans [14].

Hundreds of ingredients are added to ready-to-eat food**s** during processing to enhance the flavor of the final product. A second group of ingredients, particularly used in ready-made foods, is monosodium glutamate (MSG). As an example, although there is some belief associated with their empty calories in snack foods, they can play an important role in increasing energy consumption, via their additives such as MSG, even in early studies [15], [16]. There are reports demonstrating why it is a potent neuroexcitatory amino acid involved in several behavior patterns [17], [18]. Indeed, this compound has widespread use as a flavor-enhancing food additive and has no side effects as long as used in a safe range [19], [20]. However, studies particularly performed among children ages 12–17 demonstrated that consumption of sugar-sweetened beverages rather than salty snacks has an important impact on the development of obesity [21]. Furthermore, in several animal studies, authors demonstrated that MSG provided marked neurotoxicity on neural stem cells and hippocampal neurogenesis [22], [23] besides its known dietary differential effects on hippocampal dependent memory [24].

Both clinical and experimental studies suggest that there are two opposing views regarding whether ingredients (i.e., TAU and MSG) have adverse effects on brain health when they are used as additives in ready-to-eat foods and energy drinks. Given the concerns regarding the safety of TAU and MSG and their potential harmful effects on certain tissues, this study aimed to investigate the possible adverse effects of high levels of TAU or MSG consumption, either alone or in combination, on systemic and brain tissue oxidative stress/antioxidant balance parameters, as well as cortical brain histology.

## 2 Materials and methods

### 2.1 Materials and chemicals

Monosodium glutamate (MSG; L-glutamic acid monosodium salt hydrate; Cat. No. G1626; CAS No. 142–47–2) and taurine (Cat. No. T8691; CAS No. 107–35–7) were purchased from Sigma-Aldrich (Edinburgh, UK). The dosages of TAU and MSG administered to rats (expressed as mg/kg/day and total duration of administration) were calculated in accordance with previously published experimental studies [25], [26].

Total antioxidant status (TAS) and total oxidant status (TOS) were measured using commercial assay kits (Cat. No. TAS: RL0017; TOS: RL0024, Rel Assay Diagnostics). The levels of 4-hydroxy-2-nonenal (4-HNE) and 8-hydroxy-2′-deoxyguanosine (8-OHdG) were determined using ELISA kits obtained from Elabscience (Cat. No. 4-HNE: E-EL-0128; 8-OHdG: E-EL-0028).

### 2.2 Animal care and experimental design

**Fig. 1:**
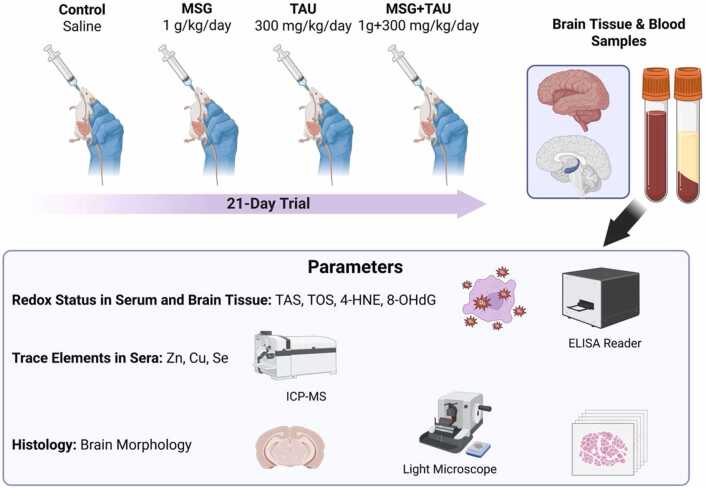
A workflow for experimental studies.

The experimental design is shown in Fig. 1. Wistar young male rats (10 weeks) were separated randomly into four groups as control rats (CON group; treated with saline, daily), monosodium glutamate treated rats (MSG group; 1 g/kg/day), taurine treated rats (TAU group; 300 mg/kg/day), and rats treated with MSG and TAU mixture (MSG and TAU group; 1 g+300 mg/kg/day). All treatments were done by oral gavage for 21 days, while every group included 7 ≥ rats.

All experimental animals were fed with a standard commercial rat chow obtained from Korkuteli Yem Gıda San. Tic. A.Ş. (Antalya, Türkiye). The diet contained soybean meal, corn, barley, wheat bran, pellet binder, dicalcium phosphate (DCP), bypass fat, toxin binder, poultry mineral premix, D, L-methionine, L-lysine, mineral mixture, and choline chloride. The nutritional composition of the diet was as follows: crude protein 24.0%, crude fat 3.14%, crude fiber 4.88%, crude ash 5.84%, lysine 1.24%, methionine 0.42%, calcium 0.88%, phosphorus 0.94%, and sodium 0.04%.

### 2.3 Sample collection

Following the 21-day experimental period, all experimental animals were anesthetized with a ketamine–xylazine combination (ketamine, 50 mg/kg; xylazine, 5 mg/kg, intraperitoneal) before sacrifice, as described elsewhere [27]. Intracardiac blood samples were collected into evacuated tubes containing gel separators (Vacutainer SST, Becton Dickinson, USA). The blood samples were allowed to clot at room temperature for 30 min and subsequently centrifuged at 1300 ×g for 15 min at 4°C to separate the serum part. The separated serum was immediately aliquoted into sterile Eppendorf tubes and stored at −80°C until further biochemical analyses were performed.

In the second part, the brain samples, such as the cortex and hippocampus, were isolated, and tissue collection was planned according to the biochemical and histopathological analysis requirements of the study. The second group of tissues was snap-frozen in liquid nitrogen and stored at −80°C until analysis.

### 2.4 Tissue homogenization

Cortex and hippocampal tissues were homogenized on ice in phosphate-buffered saline (PBS) to preserve protein integrity for ELISA and biochemical analyses. Briefly, 0.1 g of tissue was transferred into a tube and mixed with 0.9 mL of PBS. Zirconium beads were added, and the tube was tightly capped and placed in an Allsheng BioPrep-6 homogenizer. Homogenization was performed at 3,200xg for 30 s, followed by a 45 s resting period, and then repeated for an additional 30 s at the same speed. The resulting homogenates were centrifuged, and the supernatants were carefully collected and stored at −80°C until ELISA and biochemical measurements were performed.

### 2.5 Assessment of oxidative stress indicators

TAS, TOS, 4-HNE, and 8-OHdG, which are established indicators of oxidative stress and cellular injury, were measured in cortical, hippocampal, and serum samples. TAS and TOS were measured using automated colorimetric assays (Rel Assay Diagnostics, Turkey). TAS was determined based on the decolorization of a stable ABTS radical cation, while TOS was measured via oxidation of the ferrous ion–o-dianisidine complex to ferric ions forming a colored complex with xylenol orange. Results were expressed as mmol Trolox equiv./L and μmol H₂O₂ equiv./L, respectively. The oxidative stress index (OSI) was calculated as TOS/TAS after unit conversion (OSI = TOS (μmol H₂O₂ equiv./L) / TAS (μmol Trolox equiv./L). 4-HNE, and 8-OHdG levels were analyzed using commercial assay kits in accordance with the manufacturer’s instructions.

TAS and TOS levels were measured in serum, cortex, and hippocampus samples using colorimetric kits. All analyses were performed on a fully automated biochemistry analyzer (MINDRAY BS-400) in accordance with the manufacturer’s instructions. TAS measurements were conducted at an absorbance of 660 nm within a measurement range of 0.1–3.5 mmol/L, with intra- and inter-assay coefficients of variation (CVs) of 2.8% and 3.3%, respectively. TOS levels were determined at 546 nm absorbance over a range of 0.2–80 µmol/L, with intra- and inter-assay CVs of 3.2% and 3.9%. Both types of assays were based on the endpoint reaction principle. We also calculated the OSI, which expresses the ratio of TOS to TAS and reflects the level of oxidative stress in the organism [28].

Levels of 4-hydroxy-2-nonenal (4-HNE) and 8-hydroxy-2′-deoxyguanosine (8-OHdG) were quantified in serum and tissue samples using ELISA kits specific for rat samples, following the manufacturers’ protocols. Absorbance readings were obtained using a microplate reader (BIO-TEK ELx800), and washing steps were carried out with a strip washer (BIO-TEK ELx50). The 4-HNE ELISA kit exhibited a sensitivity of 0.38 ng/mL and a measurement range of 0.63–40 ng/mL, with intra- and inter-assay CVs of < 8% and < 10%, respectively. The 8-OHdG ELISA kit had a sensitivity of 0.94 ng/mL, a measurement range of 1.56–100 ng/mL, and CVs values of < 8% and < 10%. All samples were analyzed in duplicate.

### 2.6 Determination of trace element levels

For trace element analysis, blood samples were collected into trace element–free sodium heparin tubes (6 mL Vacuette® NH Trace Elements Tube, Lot No. A24073UA) supplied by Greiner Bio-One and centrifuged to obtain serum. Serum zinc (Zn), copper (Cu), and selenium (Se) concentrations were determined using Inductively Coupled Plasma Mass Spectrometry (ICP-MS) with a PerkinElmer NexION 2000 system. All analytical procedures were performed in accordance with the manufacturer’s protocols.

### 2.7 Histological examinations

Brain samples were fixed in 10% neutral buffered formalin, processed through routine paraffin embedding procedures, and sectioned at a thickness of 4–5 µm using a rotary microtome. The sections were mounted on glass slides, deparaffinized in xylene, and rehydrated through a graded series of ethanol. Hematoxylin–eosin staining was performed according to standard protocols. Following dehydration and clearing, the sections were coverslipped and examined under a light microscope for histological evaluation.

### 2.8 Statistical analysis

All statistical analyses were performed using SPSS version 27.0 software. The distribution characteristics of the data were evaluated using the Shapiro–Wilk test. Non-parametric statistical methods were applied to parameters that did not exhibit a normal distribution. Comparisons among the four groups were conducted using the Kruskal–Wallis test, followed by Bonferroni correction for multiple comparisons. Results were expressed as mean ± SEM, and p < 0.05 was considered statistically significant.

## 3 Results

### 3.1 General systemic parameters of experimental animals

In the present study, the concentration of MSG (1 g/kg/day) and TAU (300 mg/kg/day) was selected based on previously published experimental models investigating oxidative stress, excitotoxicity, and neurobiological alterations [25], [29]. In rodent studies, TAU has been commonly administered in a wide range of 25–1000 mg/kg/day, depending on the study aim, with a concentration of around 300 mg/kg/day frequently used to evaluate metabolic and antioxidant effects under increased exposure conditions [26], [30], [31], [32], [33]. Similarly, MSG has been widely used in experimental neurotoxicity and oxidative stress models at levels ranging from 0.1 to 4.0 g/kg/day, with 1 g/kg/day representing a well-established high level used to induce measurable biochemical and histopathological changes [34], [35], [36].

**Fig. 2:**
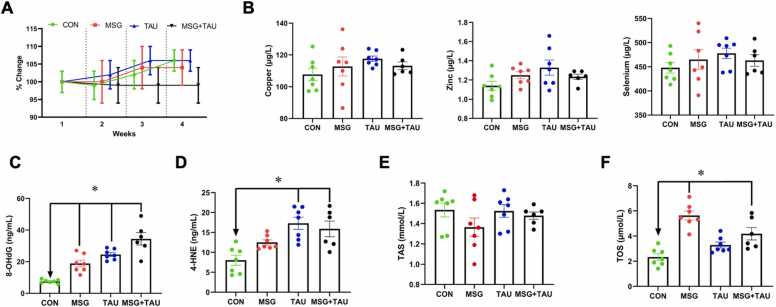
General systemic effects of treatment of male Wistar rats with nutritional ingredients such as either MSG, TAU, or their combination.

Changes in the body weight of all animals were monitored every week, and the mean values of these changes are shown in Fig. 2 (A). As can be seen in this figure, the weight gain level of either the MSG or the TAU group was very similar and parallel to that of the CON group during the experimental period (3 weeks), however, there was no weight gain in the MSG+TAU group during the same period.

The serum trace element levels of animals of all groups, such as Cu, Zn, and Se are presented in Fig. 2 (B, left, middle, right, respectively) as bar graphs (mean±SEM values of every group), and group comparisons were performed using the Kruskal–Wallis test with Bonferroni correction. These trace element levels are similar among control and experimental groups of animals, indicating no significant effect of either MSG or TAU intake, besides their combination intake on serum trace element levels for the 3-week experimental period.

### 3.2 Systemic oxidative stress status

We also determined the serum levels of two biomarkers, 8-OHdG and 4-HNE, that are measurable substances in the body used to understand what’s happening at a cellular level, especially related to oxidative stress. The mean (±SEM) level of 8-OHdG in both MSG and TAU groups is significantly higher than that of the CON group (Fig. 2C). Interestingly, the 8-OHdG level in the MSG+TAU group was significantly higher not only than that of the CON group but also higher than that of either the MSG or TAU group, as well.

Another parameter determined in the serum level of oxidative stress marker is 4-HNE. As can be seen from the bar graphs given in Fig. 2D, the serum level of 4-HNE was higher in both MSG and TAU groups, while the difference between the TAU and CON group is significant. The bars represent the mean (±SEM) values, and the group comparisons were performed using the Kruskal–Wallis test with Bonferroni correction. In addition, this level in MSG+TAU group was significantly higher than that of the CON group.

We also determined the serum TAS levels in all experimental groups compared to that of CON group. As can be seen in Fig. 2E, there are no significant differences between the mean (±SEM) values of three experimental groups and the CON group when compared with the Kruskal–Wallis test with Bonferroni correction. However, when we compare the serum TOS levels of these three groups with the CON group, there are significant differences. Interestingly, the TOS level of the MSG group is also significantly higher than that of the TAU group (Fig. 2F).

We also calculated the oxidative stress index (OSI) for the groups and then compared the experimental groups with the CON group. The OSI value (mean±SEM) of either the MSG (0.43 ± 0.04), TAU (0.22 ± 0.01), or MSG+TAU (0.30 ± 0.03) group is higher than that of the CON group (0.15 ± 0.02) when the groups are compared with the Kruskal–Wallis test with Bonferroni correction (P < 0.05). Interestingly, the highest value for significance is obtained by comparing the MSG group to the CON group (P < 0.001).

### 3.3 Histological findings in the cortical tissue of the experimental animals

**Fig. 3:**
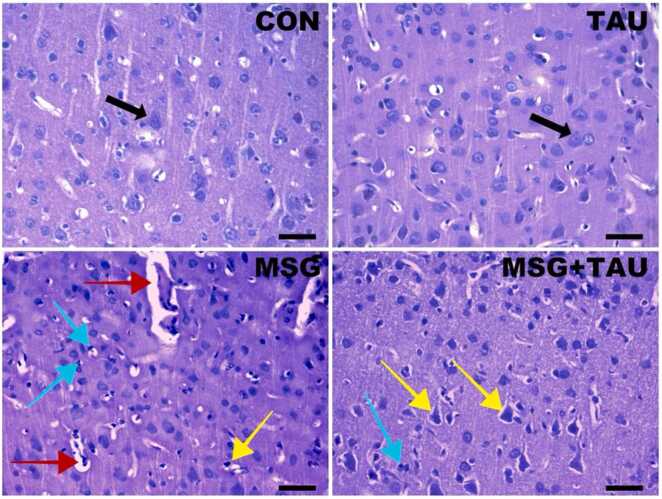
Representative histological findings in the cortical tissues of rats treated with either MSG, TAU, or their combination.

To validate the effects of systemic effects of the supplements on the brain structure, we evaluated the cortex tissue sections stained with hematoxylin and eosin (HE) and photographed using a standard light microscope. As can be seen in Fig. 3, in the MSG group, there are marked shrinkage of pyramidal neurons and nuclear pyknosis as well as slightly decreases in neuron distribution in the cortical samples. Furthermore, we observed disruption of neuronal alignment in cortical layers III (L3) and V (L5) with structural disorganization within the cerebral cortex, and pericapillary edema. In contrast, these histopathological alterations were less pronounced in the TAU group. However, these degenerative alterations in the MSG+TAU group were similar to those observed in the MSG group but were slightly more pronounced. Additionally, the CON group exhibited normal neuronal morphology, as expected.

### 3.4 Oxidative stress status in the cortical tissue

**Fig. 4:**
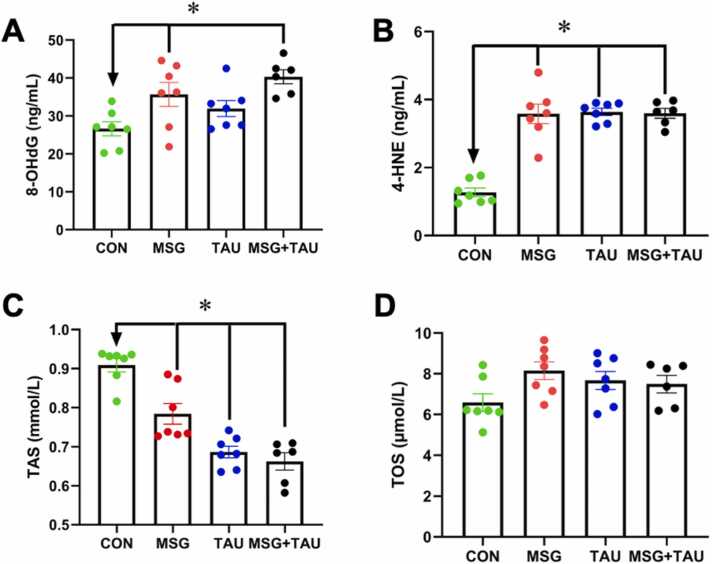
The effects of either MSG, TAU, or their combination treatment on some oxidative stress biomarkers of the cortical tissue.

We also determined the oxidative stress biomarkers in the homogenized cortical tissue sections, similar to those examined in the sera of the animals. The cortex tissue levels of 8-OHdG (as mean ± SEM) were higher in both the MSG and TAU groups compared to the CON group; however, the increase was more pronounced in the MSG group than in the TAU group (Fig. 4A). Interestingly, the significance level between the MSG+TAU and CON group is highest (P < 0.01) among the comparisons of the other two experimental groups. The second measurable substance in tissues, especially related to oxidative stress, 4-HNE level (mean±SEM), was determined almost similarly and significantly higher in these three experimental groups compared to the CON group (Fig. 4B).

We also determined the cortical tissue section TAS and TOS levels in all experimental groups compared to those of the CON group. As can be seen in Fig. 4C, there are significantly lower TAS levels (mean±SEM) in the MSG and TAU groups, as well as the MSG+TAU group, than in the CON group. When we compare the tissue TOS levels of these three groups with the CON group, there are significant differences between the three experimental groups and the CON group with a similar significance level (Fig. 4D).

We also calculated the OSI for the groups and then compared the experimental groups with the CON group. The OSI value (mean±SEM) of either the MSG (1.04 ± 0.06), TAU (1.09 ± 0.05), or MSG+TAU (1.13 ± 0.05) group is significantly higher than that of the CON group (0.74 ± 0.05) when the groups are compared with the Kruskal–Wallis test with Bonferroni correction (P < 0.05).

### 3.5 Oxidative stress status in the hippocampus tissue sections

**Fig. 5:**
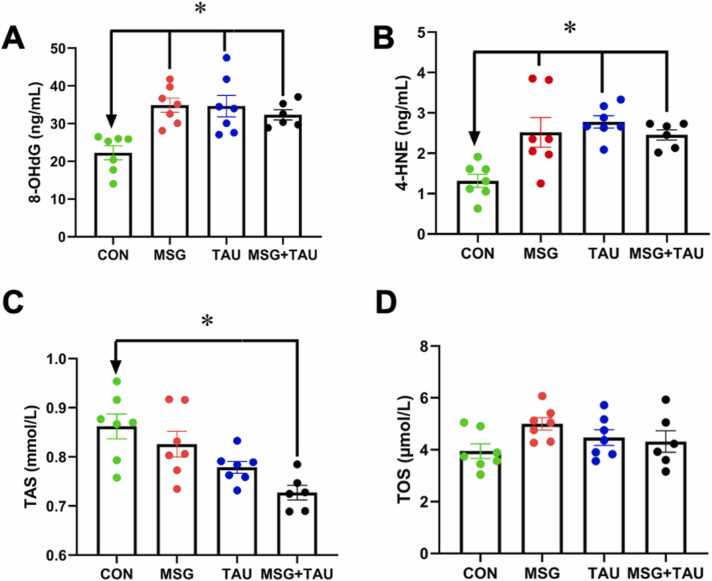
The effects of either MSG, TAU, or their combination treatment on some oxidative stress biomarkers of hippocampus tissue.

We, for further validation, determined the similar oxidative stress biomarkers in the homogenized hippocampus tissues to those of the cortical tissues. The hippocampus tissue levels of 8-OHdG and 4-HNE (mean±SEM) in these three experimental groups (MSG, TAU, MSG+TAU groups) are significantly higher than those of the CON group, while there are no significant differences among experimental groups (Fig. 5A and B, respectively).

We also determined the hippocampus tissue TAS and TOS levels in all experimental groups compared to those of the CON group. As can be seen in Fig. 5C, there are significantly lower TAS levels (mean±SEM) in the MSG and TAU groups, as well as the MSG+TAU group, than in the CON group. The lowest TAS level was determined in the MSG+TAU group compared to the CON group (P < 0.01). When we compare the tissue TOS levels of these three groups with the CON group, there are no significant differences between the three experimental groups and the CON group, as well as no significant differences among the experimental groups (Fig. 5D).

The calculated OSI for the groups (mean±SEM) of either the MSG (0.61 ± 0.03), TAU (0.57 ± 0.03), or MSG+TAU (0.60 ± 0.06) group is significantly higher than that of the CON group (0.46 ± 0.04) when the groups are compared with the Kruskal–Wallis test with Bonferroni correction (P < 0.05). There are no significant differences among these three experimental groups.

## 4 Discussion

Food ingredients generall are used to help make ready-to-eat foods tasty, safe, convenient, nutritious, and long-lasting. However, some food ingredients can be harmful to human health, but this depends on the type of ingredient, the amount consumed, and how often it is consumed [37], [38], [39]. In this context, MSG and TAU are among the most frequently studied dietary-related compounds due to their widespread use and potential physiological effects. Experimental and some preclinical studies have shown that TAU intake has increased over the last half century due to increased consumption of TAU-containing energy drinks and dietary supplements. It remains unclear whether long-term intake of high level of TAU induces cellular adaptations via affecting various molecular pathways. Therefore, in the present study, we aimed to demonstrate the possible toxic effects of high level intake of either MSG, TAU or their combination in mammalian body besides spesific tissues.

The earliest widely cited scientific article that clearly discusses the role of TAU in humans and presents the physiological actions and functions, including its metabolism, bile salt conjugation, osmoregulation, and cellular Ca^2 +^ homeostasis [40]. Indeed, it is abundant in various tissues such as heart, brain, and retina with relevance to human health [11]. Additionally, the sodium salt of glutamic acid, MSG, has basically induced a unique taste called umami and makes food palatable in foods [41]. However, it remains unclear whether long-term high-level exposure to these ingredients induces cellular remodeling affecting the redox balance and related molecular pathways in mammals. Thus, here, we focused on investigating the effects of high-level supplementations of either MSG, TAU, or their combination to demonstrate their potential deleterious effects on certain tissues, by analyzing the structure and the biochemical parameters of the brain tissues, besides the serum oxidative stress status as systemic parameters. Following a 3-week supplementation of the male rats, there was no body weight gain in the animals from the MSG+TAU group during the supplementation period, while no effect with alone MSG or TAU supplementation on the body weight gain levels of the animals. This finding can point out an induction of an abnormal weight gain process in the animals supplemented with two ingredients when the animals consumed over their normal consumption levels. Furthermore, when we analyzed the systemic parameters of the animals by using some redox balance parameters in sera, the most attractive changes were determined in animals consuming these two ingredients together. Parallel to the systemic effects of these ingredients, the brain oxidative stress parameters as well as the histology findings strongly supported the toxic effects of these ingredients when they consumed over their daily intake levels. When we consider our overall data in the present study, our present findings strongly support the possible adverse effects of a combined supplementation of MSG and TAU for 3 weeks, in part, through an increase in both systemic and brain oxidative stress status with significant depression in the antioxidant status of the animals which are leading to altered level of redox balance in the system.

Literature data have provided various information about why these types of ingredients can present deleterious effects in the mammalian body [42]. Although TAU plays a beneficial role in diverse metabolic and physiological processes, such as glucose and lipid regulation, energy metabolism, anti-inflammatory modulation, and antioxidant actions [43], it can induce some toxicity when it is consumed at over-normal levels, particularly together with caffeine, sugar, and alcohol [44], [45]. Despite the antioxidant and neuroprotective effects of TAU and caffeine from energy drinks, their excessive consumption among adolescents can lead to various disorders, such as high systolic blood pressure, agitation, anxiety, heart palpitations, and poor sleep quality [46], [47]. Supportingly, another experimental study suggest that a high level of TAU can provoke behavioral sensitization, indicating central effects of chronic TAU in rats, suggesting that the amino acid has some behavioral properties or that only a certain portion of peripherally available TAU enters the brain [48]. Importantly, it has been emphasized that both *in vitro* and *in vivo* studies have shown active transport of TAU across the blood brain barrier into the brain [49], [50] and across the blood-CSF barrier [49], [51]. However, it has been also point out that more research is warranted to define the signaling systems involved related with the effects of TAU consumption. In addition, MSG is the most commonly used food additive and has well-known neurotoxic effects [52]. Authors in this study found that MSG consumption presented important abnormalities in behavioral activities, as well as alterations in biochemical and immunohistochemical parameters of the hippocampus in male rats. Although there are several pathways defined related to the effect of MSG on either tissue, function, or cell level, the most defined pathway seems to be related to redox status. Others also mentioned that MSG treatment of rats induced notable variations in behavioral performance and exerted excitotoxic effects on the brain, leading to significant impairment in short-term memory and alterations in exploratory behavior in rats [53]. The underline mechanisms of these effects with MSG treatment have been proposed to be associated with its excitotoxic impacts via glutamate interaction with its receptors, promoting apoptosis and neuronal cells [54]. In addition, other studies suggested that MSG could induced alterations in memory function and reduced the generation of pro-inflammatory cytokines in the cortex and hippocampus of mouse brains [55]. Also, the findings of these studies have shown that rats treated with MSG had significantly enhanced glucose, cholesterol, and triglycerides levels [56], [57]. There are also other animal studies examined the potential toxic effect of MSG on neurons in various regions of the hippocampus in prepubertal rats [58]. Overall interpretation our present data together with previously published data, it can be concluded that either TAU or MSG or their combination can induce some but important deleterious actions in brain tissues, particularly when consumed high levels, which can further lead to functional abnormalities.

In conclusion, in the present study, high-level supplementation alone of either MSG or TAU, or their combination, to animals for a chronic manner produced deleterious effects in serum parameters as well as brain tissue parameters, while the histological alterations in cortical samples were parallel to the biochemical alterations. Furthermore, both biochemical and histological analysis results of brain tissue samples strongly imply that any exposure to excessive intake of either MSG or TAU can alter redox balance and integrity of brain tissue with varying degrees, while their combined exposure produces more pronounced deleterious effects. Overall, these findings suggest that frequent consumption of foods and beverages containing MSG and TAU, particularly at high levels, may pose significant health risks to young individuals.

Taking these experimental results into consideration, it can be suggested that MSG or TAU, either alone or in combination, may influence redox balance pathways and the neuronal structure of the brain in a manner dependent on exposure concentration and duration. However, the present study was conducted using a high-level experimental model, with exposure levels exceeding typical human dietary intake; therefore, extrapolation of these findings to normal dietary conditions should be made with caution.

## Ethics Statement

The rats and experimental procedures were conducted in accordance with protocols approved by the Ankara University Experimental Animals and Research Laboratory (DEHAL), and the ethical approval was obtained from the Ankara University Animal Experimentation Local Ethics Committee (Decision number: 2025–13–148 at 09.07.2025). No human participants were involved in this study.

## Funding

This study was supported by the Scientific Research Projects Coordination Unit of Lokman Hekim University with Project No. TSA-2025–130. Grant holder is Assist. Prof. Dr. Yasemin Atıcı.

## CRediT authorship contribution statement

**Dogan Yucel:** Writing – review & editing, Supervision. **Belma Turan:** Writing – review & editing, Writing – original draft, Supervision. **Atakan Saglam:** Formal analysis, Data curation. **Gamze Dogan:** Formal analysis, Data curation. **Makbule Beyza Sen:** Investigation, Formal analysis, Data curation. **Yasemin Atici:** Writing – review & editing, Visualization, Validation, Project administration, Methodology, Investigation, Funding acquisition, Formal analysis, Data curation, Conceptualization.

## Declaration of Competing Interest

The authors declare that they have no known competing financial interests or personal relationships that could have appeared to influence the work reported in this paper.

## Data Availability

The data that has been used is confidential.
